# Xywav: A Treatment Option for Idiopathic Hypersomnia in Down Syndrome

**DOI:** 10.7759/cureus.89339

**Published:** 2025-08-04

**Authors:** Serena Sanchez, Julie Allen

**Affiliations:** 1 Internal Medicine, White County Medical Center, Searcy, USA; 2 Sleep Medicine, Searcy Medical Center, Searcy, USA

**Keywords:** down syndrome, idiopathic hypersomnia, oxybates, sleep disorders, xywav

## Abstract

Idiopathic hypersomnia (IH) is a rare and chronic neurological sleep disorder in which patients experience excessive daytime sleepiness (EDS) with symptoms often overlapping with numerous other sleep conditions. This diagnostic overlap can further delay accurate diagnosis and treatment, which poses significant challenges for both patients and clinicians. Additionally, vulnerable populations, such as those with Down syndrome (DS), face further obstacles given the absence of therapeutic studies addressing IH in DS. This case presentation highlights the case of a 40-year-old female patient with DS who suffered from substantial hypersomnolence and had responded favorably to a supervised trial of Xywav (calcium, magnesium, potassium, or sodium oxybates), making this the first known case presentation to demonstrate an effective treatment strategy for this population.

## Introduction

Idiopathic hypersomnia (IH) is a rare and chronic neurological sleep disorder in which patients experience excessive daytime sleepiness (EDS) despite sleeping a normal or longer-than-normal amount of sleep time [1]. A multitude of debilitating symptoms can present and persist in IH, including EDS, increased sleep inertia, unrefreshed naps, prolonged sleep time, and cognitive impairment [2]. The pathophysiology of IH remains unclear, with proposed mechanisms including genetic predisposition, inflammatory or immune dysfunction, or dysregulation of GABAergic signaling [2,3], though conclusive evidence is lacking. Unlike narcolepsy type I (NT1), which is grounded in the loss or dysfunction of orexin (i.e., hypocretin) along with a genotypic association, the condition presents with characteristic symptoms such as EDS, cataplexy, hallucinations, and sleep paralysis [4]. However, with IH, there are no specific biomarkers, and because the symptoms overlap with numerous other sleep conditions, the differential diagnosis and subsequent treatment can be extremely challenging [5].

The prevalence of IH varies due to a suspected underdiagnosis and overlap with other sleep conditions. This is exacerbated by the relatively low awareness of the condition, which can further prolong the diagnosis by up to 10-15 years, with nearly 47% of patients being labeled narcoleptic even before their first IH diagnosis [6]. Currently, an estimated 37,000 patients in the United States are actively seeking care for IH; however, due to likely underdiagnosis, it is suggested that the true prevalence may be greater [7]. 

The former pharmacological options for treating IH have primarily relied on off-label approaches; however, as additional research has continued, modafinil (Provigil) has become the first-line option and was supported with the strongest recommendation [5]. Treatment with fewer recommendations included methylphenidate, amphetamines, pitolisant, clarithromycin, mazindol, and flumazenil [5]. However, in August 2021, the United States Food and Drug Administration (FDA) approved Xywav, which contains calcium, magnesium, potassium, or sodium oxybates, for the treatment of IH; it became the only approved treatment, thereby offering an updated approach to managing this condition [7]. 

The exact mechanism of action of Xywav is unknown, but it is hypothesized that it acts through GABA-B receptors at dopaminergic, nonadrenergic, and thalamocortical neurons, thereby modulating GABAergic neurotransmission, which subsequently leads to improved EDS and sleep architecture [7]. Further, the implementation of Xywav has revealed that over 80% of participants have achieved clinically meaningful responses on the Epworth Sleepiness Scale (ESS) and Idiopathic Hypersomnia Severity Scale (IHSS) with up to 95% (ESS) and 98% (IHSS) showing decreases of ≥ 4 points at the end of the study [8]. 

No known clinical trials or published studies have evaluated the use of Xywav in individuals with DS. This highlights the need for further research to establish the safety and efficacy of using Xywav in the treatment of IH in individuals with DS. Therefore, this report contributes to the literature by describing a case in which Xywav was utilized and highlights the importance of exercising caution while considering the unique physiological and neurological attributes of DS in the treatment of IH.

## Case presentation

The patient, a 40-year-old high-functioning, articulate female, had a complex medical history. She was diagnosed with DS, orthostatic hypotension, renal osteodystrophy, hypothyroidism, scoliosis, obsessive-compulsive disorder (OCD), and obesity. She presented to the sleep clinic in 2023 with her supportive mother, who endorsed a lifelong affliction of daytime hypersomnolence, which significantly worsened in 2001. 

In 2002, the patient was initially diagnosed with mild obstructive sleep apnea (OSA) with a respiratory disturbance index (RDI) of 9.6 per hour. An overnight titration was performed, and she was subsequently started on continuous positive airway pressure (CPAP) therapy at 6 cmH_2_O. CPAP efficacy was unclear, as hypersomnolence persisted despite therapy. In May 2004, the patient underwent another split-night sleep study with an RDI of 5.3 and an adjustment of her positive airway pressure (PAP) pressure to 10 cmH_2_O. 

In September 2009, a repeat overnight polysomnography (PSG) revealed an apnea-hypopnea index (AHI) of 1.9, which was consistent with normal sleep-related breathing or no overt sleep apnea. As the patient continued to experience hypersomnolence, a next-day multiple sleep latency test (MSLT) was performed, which included a negative urine drug screen and five nap trials. The test revealed a short sleep latency of three minutes and 48 seconds, with one sleep-onset rapid eye movement (SOREM) period. While reviewing her history, for unknown reasons, she was continued on PAP therapy. Further, her PSG was read as possible narcolepsy, and consequently, the patient was started on Nuvigil (armodafinil) and Provigil (modafinil) for narcolepsy without cataplexy. 

In 2011, the patient remained on nightly PAP therapy, despite a former AHI of 1.9, and despite adherence, the patient continued to experience hypersomnolence. In August 2011, CPAP therapy was discontinued, and overnight pulse oximetry revealed oxygen saturations of greater than 90%. The mother reported improved sleep quality and reduced hypersomnolence after CPAP discontinuation, suggesting therapy may have disrupted sleep. The patient remained on Nuvigil (armodafinil) 150 mg, ½ tab up to twice daily, but was weaned off as the family felt her symptoms remained consistent with or without the use of medication.

In 2014, she had a significantly elevated ESS of 20. At that time, she was following a psychologist for possible excessive compulsive disorder and was prescribed clomipramine 150 mg nightly. However, as the medication impacts rapid eye movement (REM) sleep, an MSLT was not performed. A repeat PSG occurred in September 2014, which revealed an AHI of 4.5, suggesting a regular sleep-related breathing pattern, oxygen saturation above 90, and no indication for PAP therapy. The PSG showed periodic limb movements (PLM) of 7.3 associated with arousals that were minimally visible on video and unlikely to contribute to continued daytime hypersomnolence. It should be noted that clomipramine could also lead to increased periodic limb movements at night. The patient’s iron studies revealed no abnormalities suggestive of restless leg syndrome (RLS). Clomipramine was continued. The patient maintained a consistent sleep schedule, with a 9:00 a.m. awakening, a midnight bedtime, and a 30-minute sleep latency. At that time, her treatment regimen included medications for hypersomnia and narcolepsy without cataplexy based on her continued symptoms and previous MSLT findings. Despite remaining on Provigil (modafinil) 200-400 mg daily and Nuvigil (armodafinil) up to 250 mg daily, this provided only marginal relief of her daytime hypersomnolence. Further, the mother noticed increased compulsiveness and talkativeness while on these medications. Subsequent trials with Ritalin (methylphenidate) and Adderall (amphetamine/dextroamphetamine) did not yield significant improvement in her symptoms. Due to continued daytime hypersomnolence, the patient underwent surgical evaluation for an Inspire device (Inspire Medical Systems, Inc., Minnesota, United States). This hypoglossal nerve stimulator acts to retract airway muscles and maintain a patent airway during sleep [9]. Ultimately, the patient did not undergo Inspire placement but instead underwent a tonsillectomy, given 4+ lingual tonsils, along with an epiglottidectomy and palatal stiffening.

In September 2020, another PSG revealed an AHI of 78 and oxygen saturation of 74%, indicating a significant increase in her sleep apnea, consistent with severe OSA. The patient’s sleep latency remained shortened at three minutes with PLM at 0. The patient was placed back on auto-positive airway pressure (APAP) therapy. 

In 2023, the patient presented to our outpatient sleep clinic with an endorsed ESS score of 16-20. The patient maintained a consistent sleep schedule, with a 9:00 a.m. awakening and a midnight bedtime. The mother relayed that the patient was PAP compliant, appeared to be sleeping well, and denied snoring. The patient was also maintaining a healthy lifestyle, consisting of protein-rich bowls, smoothies, and low-carbohydrate meals. Despite adherence, the patient continued to experience daytime hypersomnolence, and repeat laboratory tests were unremarkable.

An extensive review of her prior medical history was conducted, which revealed that cataplexy, sleep paralysis, or hallucinations had never been documented. Therefore, based on continued symptoms along with prior sleep results, the diagnosis of idiopathic hypersomnia (IH) was confirmed as the diagnostic criteria, according to the International Classification of Sleep Disorders, Third Edition (ICSD-3), were satisfied [10]. Specifically, ICSD-3 diagnostic criteria A-F must be met, which includes: (i) The patient has daily periods of irreversible need to sleep or daytime lapses into sleep occurring for at least three months, (ii) Cataplexy is absent, (iii) An MSLT performed according to standard technique shows fewer than two sleep onset REM periods or no sleep onset REM periods if the REM latency on the preceding polysomnogram was less than or equal to 15 minutes, (iv) The presence of at least one of the following: (a) The MSLT shows a mean sleep latency of ≤ 8 minutes and (b) Total 24-hour sleep time is ≥ 660 minutes (typically 12-14 hours) on 24-hour polysomnographic monitoring (performed after correction of chronic sleep deprivation), or by wrist actigraphy in association with a sleep log (averaged over at least seven days with unrestricted sleep), (v) Insufficient sleep syndrome is ruled out (if deemed necessary, by lack of improvement of sleepiness after an adequate trial of increased nocturnal time in bed, preferably confirmed by at least a week of wrist actigraphy), and (vi) The hypersomnolence and/or MSLT findings are not better explained by another sleep disorder, other medical or psychiatric disorder, or use of drugs or medications [10].

The patient and family denied any additional testing at that time. It was relayed that prior stimulant medications exacerbated her OCD and had, incidentally, been titrated off of clomipramine before she arrived at our clinic. The patient and her family relayed their frustrations due to a lack of therapeutic response, along with continued hypersomnia. 

Given the patient’s new diagnosis of IH along with continued hypersomnolence, a trial of Sunosi (solriamfetol) was attempted, which provided some improvement compared to Provigil or Nuvigil. However, the patient’s afternoon hypersomnolence was still significantly impacting her quality of life, and the family continued to inquire as to other medication options. Given that multiple medications failed to address the patient's symptoms, an alternative non-amphetamine was explored so as not to exacerbate behavioral changes. Xywav was selected and was cautiously discussed, given the patient’s cognitive functioning and DS. After a thorough literature review with minimal findings specific to this patient population, the family agreed to proceed with a slow titration as the benefits outweighed the risks.

The patient was titrated under strict supervision, and provider-family communication was frequent through phone and/or the patient portal. The patient’s titration started at 3 g nightly in week 1, increased to 4.5 g nightly in week 2, and to 6 g nightly in week 3, and continued at 6 g nightly thereafter. In 2025, the patient has remained on 6 g nightly, mixed with water at bedtime, and securely stored in a medication lock box with an alarm to remind her of her dosing schedule. The once nightly dosing was decided upon out of safety concerns, to decrease the risk of cognitive confusion, and mitigate the burden of her family having to awaken the patient for a second dose. Following Xywav initiation, daytime naps decreased, Sunsoi use reduced, and her overall disposition and hypersomnolence improved. The patient was able to discontinue her stimulants altogether and transitioned to Sunsoi as needed. Further, the patient’s blood pressure and ESS were the lowest they have been recorded since 2002. Table 1 demonstrates pertinent sleep measurements before and after Xywav use.

**Table 1:** Sleep measurements before and after Xywav use ESS: Epworth Sleepiness Scale; PAP: positive airway pressure; N/A: not applicable; AHI: apnea-hypopnea index

Year	AHI	Blood Pressure	ESS	PAP	Xywav
2002	9.6	Unknown	Unknown	Yes	N/A
2004	5.3	Unknown	Unknown	Yes	N/A
2009	1.9	115/72	18	Yes	N/A
2011	Unknown	126/76	20	No	N/A
2014	4.5	105/62	20	No	No
2020	78	Unknown	Unknown	Unknown	No
2023	4.9	100/60	16	Yes	No
2025	5.8	96/54	9	Yes	Yes

Since utilizing Xywav, either during titration or while on her single nightly dosing, she did not experience any noted side effects. The patient rarely experienced symptoms associated with RLS and chose not to take medication, given the rarity of such instances. Routine laboratory testing confirmed that there were no underlying inflammatory or metabolic concerns related to continuing the medication. The continued combination of Xywav, along with lifestyle management, contributed to a notable improvement in the patient’s sleep quality and daytime functioning, thereby allowing the patient a significantly improved quality of life. 

## Discussion

The diagnosis, along with subsequent treatment of IH, poses numerous challenges, which may prolong appropriate treatment, thereby impacting patient health and satisfaction. When vulnerable populations with varying physiological and neurological considerations, such as those with DS, need to be taken into account, this further compounds the diagnosis and treatment options available. Individuals with DS tend to have a higher prevalence of sleep-disordered breathing [11] and, therefore, more consideration should be given in the literature to the safe exploitation of IH management strategies in those with DS.

For instance, when encountering a patient with DS in clinical practice, a more detailed sleep and behavioral history, anatomic assessment, medication review, and documentation of cognitive baseline and functioning should be performed to aid with overcoming potential barriers interfering with treatment. Further, after initiating treatment, evaluations of behavioral exacerbations and caregiver education with involvement are key to ensuring a safe and appropriate response. 

The current patient underwent multiple diagnostic evaluations, including PSGs and MSLTs, as well as various pharmacological treatments aimed at addressing her daytime hypersomnolence. Despite initial symptoms being attributed to mild OSA, the persistence of hypersomnolence despite CPAP compliance and stable overnight oxygenation continued to lean toward an alternative diagnosis. Specifically, IH was consistent with ICSD-3 diagnostic criteria, given the patient’s history and exclusion of narcolepsy features. 

Xywav, a mixed-salt oxybate, is hypothesized to modulate GABA-B receptors, which leads to improved EDS and sleep architecture [7]. In this case, the once-nightly dosing with slow titration and under direct supervision was chosen to mitigate cognitive functioning and avoid mid-sleep redosing, thereby minimizing sleep disruption, all while considering the patient’s neurodevelopmental condition. 

It should be noted that a lumbar puncture to obtain orexin (i.e., hypocretin) levels was discussed but deemed too difficult due to the increased risk associated with the patient’s DS and scoliosis. Additionally, an HLA-DQB1*06:02 allele [12], which is strongly associated with narcolepsy, was not gathered as it was deemed that it would not change the course of treatment. 

Overall, the patient’s response to Xywav was notable, with improvements in the ESS, stimulant discontinuation, and a reduction in the use of another wakefulness-promoting agent, thereby improving sleep inertia and reducing hypersomnolence. The successful titration revealed no noticeable adverse effects, even with continued maintenance, indicating that Xywav may be an effective treatment option for individuals with DS experiencing IH. However, a gap remains in the medical literature regarding its use in the DS population. Therefore, further research is needed, and larger, controlled studies should be considered to validate the findings.

## Conclusions

This case demonstrates the complexity of diagnosing and treating IH in both the general population and individuals with DS. It also highlights the potential use of Xywav as a treatment option for another select group of patients. With careful titration and monitoring, Xywav has allowed for a substantial improvement in hypersomnolence and daytime functioning, thereby contributing to an enhanced quality of life for the patient and her family. This case presentation highlights the need for further research on Xywav's safety, dosing, and long-term effects in the DS populations. Overall, future investigations are needed to better guide sleep clinicians in managing IH in individuals with DS.
